# Treating Influenza Infection, From Now and Into the Future

**DOI:** 10.3389/fimmu.2018.01946

**Published:** 2018-09-10

**Authors:** Sophia Davidson

**Affiliations:** Inflammation Division, The Walter and Eliza Hall Institute of Medical Research, Parkville, VIC, Australia

**Keywords:** influenza, therapeutics, treatment, antiviral, immunomodulation

## Abstract

Influenza viruses (IVs) are a continual threat to global health. The high mutation rate of the IV genome makes this virus incredibly successful, genetic drift allows for annual epidemics which result in thousands of deaths and millions of hospitalizations. Moreover, the emergence of new strains through genetic shift (e.g., swine-origin influenza A) can cause devastating global outbreaks of infection. Neuraminidase inhibitors (NAIs) are currently used to treat IV infection and act directly on viral proteins to halt IV spread. However, effectivity is limited late in infection and drug resistance can develop. New therapies which target highly conserved features of IV such as antibodies to the stem region of hemagglutinin or the IV RNA polymerase inhibitor: Favipiravir are currently in clinical trials. Compared to NAIs, these treatments have a higher tolerance for resistance and a longer therapeutic window and therefore, may prove more effective. However, clinical and experimental evidence has demonstrated that it is not just viral spread, but also the host inflammatory response and damage to the lung epithelium which dictate the outcome of IV infection. Therapeutic regimens for IV infection should therefore also regulate the host inflammatory response and protect epithelial cells from unnecessary cell death. Anti-inflammatory drugs such as etanercept, statins or cyclooxygenase enzyme 2 inhibitors may temper IV induced inflammation, demonstrating the possibility of repurposing these drugs as single or adjunct therapies for IV infection. IV binds to sialic acid receptors on the host cell surface to initiate infection and productive IV replication is primarily restricted to airway epithelial cells. Accordingly, targeting therapies to the epithelium will directly inhibit IV spread while minimizing off target consequences, such as over activation of immune cells. The neuraminidase mimic Fludase cleaves sialic acid receptors from the epithelium to inhibit IV entry to cells. While type III interferons activate an antiviral gene program in epithelial cells with minimal perturbation to the IV specific immune response. This review discusses the above-mentioned candidate anti-IV therapeutics and others at the preclinical and clinical trial stage.

## Introduction

Influenza viruses (IVs) are a continual and re-emerging threat to human health. Annual epidemics infect approximately 1 billion individuals, leading to three to five million cases of severe illness and up to half a million fatalities worldwide (1, 2). Influenza A Virus (IAV), Influenza B Virus (IBV) and Influenza C Virus (ICV) are all members of the *Orthomyxoviridae* family. IV genomes are segmented, which allows for reassortment within, but not between, family groups. Although IBV and ICV do cause disease in humans (IBV being responsible for approximately 25% of seasonal influenza infections) IAV strains are responsible for the majority of human infections and are most likely to cause severe disease. IAV are further classified into subtypes based on the antigenic properties of two viral surface glycoproteins, hemagglutinin (HA) and neuraminidase (NA), to date 18 HA (H1–H18) and 10 NA (N1–N10) antigenic subtypes been identified (3, 4). Unlike IBV and ICV, IAV infects a broad range of species including humans, pigs, horses, wild mammals, and birds (5). Due to different preferences for sialic acid moieties direct zoonosis of IAV between birds and humans is rare, however when it does occur, the mortality rate is staggeringly high, approximately 60% for H5N1 and 30% for H7N9 (6). In worrying contrast, transmission of IAV strains from swine to humans is common (7).

In healthy humans, IV infection induces a robust immune memory response, in spite of this the average adult will experience two IV infections per decade throughout their lifetime (8). IVs are able to evade IV-specific host immunity through two mechanisms: antigenic drift and shift. Antigenic drift occurs as IV genomes do not have RNA proofreading enzymes and consequently, point mutations accumulate in the genome through successive replication. This leads to alterations in the appearance of viral antigens and eventual emergence of new IV strains which are unrecognizable to pre-existing host immunity (9). Significantly more dramatic and, within the *Orthomyxoviridae* family, believed to be specific to IAV is antigenic shift. Infection of a single host cell with two or more strains of IAV results in the reassortment of genomic segments. IAV genome segments are packaged into viral particles by the host cell without respect to the original strains, leading to progeny virions which possess new HA and/or HA and NA proteins, such as those of avian or swine origin, but may retain the ability to effectively infect humans. Antigenic shift gives IAV pandemic potential, indeed it is thought that the majority of pandemics of the Twenty-First century have been caused by reassortment events that resulted in avian or swine IAV being able to stably infect humans (10).

The severity of IV induced disease is a function of the interplay between viral virulence and the host immune response. In a mild infection the inflammatory response is controlled and cleared rapidly. However, in highly pathogenic IV infections the host immune response can become excessive. Termed the cytokine storm, severe IV infection in humans is characterized by aberrant cytokine and chemokine responses that associate with infiltration of inflammatory cells, particularly monocytes and neutrophils. This inflammation coincides with destruction of the epithelial layer and consequently, respiratory dysfunction or acute respiratory distress syndrome (ARDS) (11). *Ex vivo* analysis of clinical samples, experimental infection models and clinical trials all indicate that the cytokine storm positively correlates with tissue injury and severe IV induced disease (12–17).

To add to the multifarious nature of IV infection, it can be complicated by secondary bacterial infection. Bacteria which normally colonize the upper respiratory tract such as *Streptococcus pneumoniae* or *Staphylococcus aureus* can cause pneumonia and septicaemia in IV infection (18). It is thought that opportunistic bacteria take advantage of changes in the pulmonary environment wrought by IV infection. Many mechanisms have been proposed to explain this phenomenon, for example IV infection induces a robust type I interferon (IFNαβ) response, which blocks the recruitment of neutrophils, a cell type particularly important for clearance of bacterial infection (19). Furthermore, monocytes and monocyte-derived cells recruited to the lung during IV infection induce the apoptosis of airway epithelial cells via TNF-related apoptosis-inducing ligand (TRAIL), this facilitates bacterial colonization and systemic dissemination by compromising epithelial layer ntegrity (20).

Undeniably, there is a real and present need for effective broad spectrum anti-IV therapies. Given the high mutagenicity of the IV genome vaccine development is fraught with difficulty, current IV vaccines are strain specific and therefore a new immunization is required for each new season (21). Moreover, the rapid emergence of the 2009 H1N1 pandemic strain demonstrated how under prepared we are for a serious IAV pandemic. This review reports current treatments for IV and discusses new therapies at clinical or pre-clinical stage. As IAV has pandemic potential and is most likely to cause severe disease in humans many of the treatments discussed are primarily directed at this virus, however they may be effective against other *Orthomyxoviridae* family members. For clarity, therapies are categorized based on point of action in IV infection, specifically, (1) IV: proteins and genomes, (2) Host immune response: cytokines/chemokines and other inflammatory modulators, and (3) Target cells for IV replication: respiratory epithelium.

## Direct targeting of IV

### Current treatment

IV surface proteins HA and NA are responsible for virion attachment to and detachment from sialic acid moieties on the host cell surface. HA attaches to cell surface sialic acid receptors to initiate viral entry and promote fusion of viral and cellular membranes, while NA acts as a sialidase, cleaving the α-ketosidic bond linking a terminal neuraminic acid residue to the adjacent oligosaccharide moiety. This enzymatic action of NA releases IV particles from infected cells and thereby allows the spread of IV to naive cells (22). NA sialidase activity also facilitates the movement of IV through the sialic acid-rich mucous of the human respiratory tract (23). NA is essential for productive IV infection and the catalytic sites of NA are conserved across IAV and IBV strains, making this glycoprotein an attractive target for antiviral therapy (24). Accordingly, in the 1990s Neuraminidase inhibitors (NAIs) were developed. NAIs are sialic acid analogs which competitively bind to the active site on NA molecules to inhibit the release of IV progeny from the cell surface (25).

NAIs are the only antivirals currently recommended to treat IV infection, oseltamivir and zanamivir are used worldwide, laninamivir is approved in Japan and peramivir is approved in China, Japan, South Korea, and the United States (26). Oseltamivir (Table 1) is most commonly used and has been shown *in vitro* to have activity against human and avian IAV subtypes and IBV strains (27). NAIs have been employed successfully for over decade, however between 2007 and 2009 resistance to oseltamivir in seasonal IAV strains surged from less than 1% to over 90% (28–31). IV strains resistant to NAIs typically contain mutations in the NA which reduce the inhibitor binding ability by altering the shape of the NA catalytic site. Although several resistance conferring mutations have been reported, the most common for IAV is H274Y. In order for oseltamivir to bind correctly, NA must undergo rearrangements to form a binding pocket. Key to these rearrangements, is the amino acid E276 rotating and binding to R224 (32, 33). *In vitro* modeling and X ray crystallography revealed that H274Y inhibits this rotation of the E276 residue thereby preventing pocket formation (32, 34). Such a dramatic uptake of the H274Y mutation at the population level is unlikely to be driven by individual patient use, instead H274Y-mutant IAV strains may have acquired advantageous epidemiologic fitness, allowing for rapid global transmission (35, 36). Fortunately, the 2009 H1N1 IAV pandemic strain did not carry this mutation when it emerged, and as this is the current dominant seasonal strain, the frequency of NAI resistance in circulating IAV strains is now low. However, localized clusters of oseltamivir-resistant IAV have been detected (37), and mutations which confer decreased sensitivity to oseltamivir in IBV strains have also been reported (38). The rapid emergence of oseltamivir-resistance observed between 2008 and 2009 demonstrates that NAI-resistance can develop at no cost to viral fitness and these mutations can easily spread throughout the population.

**Table 1:** Summary of key treatments discussed.

	**Therapy**	**Stage**	**Activity**	**Specificity**	**Effect on IV replication**	**Effect on host inflammatory response**	**Effect on epithelial Cells**	**Viability**
IV	MHAA4549A	Phase II	Antibody to HA stem region, induces cellular cytotoxicity of infected cells	IAV only	Inhibitory	Inhibitory	Not reported	Good
	MEDI8852	Phase II	Antibody to HA stem region, induces cellular cytotoxicity of infected cells	IAV only	Inhibitory	Not reported	Not reported	Good
	VIS-410	Phase II	Antibody to HA stem region, induces cellular cytotoxicity of infected cells	Select IAV strains	Inhibitory	Not reported	Not reported	Good
	JNJ63623872	Phase II	Inhibits IV replication by binding PB2 and preventing 7-methyl GTP docking	IAV only	Inhibitory	Not reported	Not reported	Moderate
	Favipiravir	Approved/phase II	Inhibits generation of viable IV particles by driving mutations in IV genome	None	Inhibitory	Not reported	Not reported	Good
	JJ3297	Preclinical	Inhibits NS1 activity	None	Inhibitory	Stimulatory	Not reported	Unknown
Immune response	Etanercept	Clinically approved for other	TNF receptor decoy, blocks TNFα signaling	None	Inhibitory	Inhibitory	Not reported	Unknown
	IFNαβ	Clinically approved for other	Induces expression of antiviral and inflammatory genes in epithelial cells and immune cells	None	Inhibitory	Stimulatory	Increased cell death	Low
	AAL-R	Preclinical	Inhibits inflammatory cytokine and chemokine secretion and immune cell recruitment by agonism of S1PRs: 1, 3, 4, and 5	None	No effect	Inhibitory	Decreased cell death	Low
	CYM-5442	Preclinical	Inhibits inflammatory cytokine and chemokine secretion and immune cell recruitment by agonism of S1PR1	None	No effect	Inhibitory	Decreased cell death	Moderate
	RP-002	Preclinical	Inhibits inflammatory cytokine and chemokine secretion and immune cell recruitment by agonism of S1PR1	None	No effect	Inhibitory	Decreased cell death	Moderate
	Celecoxib	Clinically approved for other (Phase III for IV)	COX-2 inhibitor, may blunt immunopathology through induction of PGE_2_	None	No effect	Inhibitory/no effect	Not reported	Moderate
	Statins	Clinically approved for other	Competitive inhibitors for HMG-CoA reductase, blunts inflammation and viral replication in some settings	None	Inhibitory	Inhibitory	Not reported	Moderate
	Pioglitazone	Clinically approved for other	PPARγ agonist, decreases recruitment of tipDCs	None	No effect	Inhibitory	Not reported	Moderate
Epithelial cells	Fludase	Phase II	Removes IV entry point into epithelial cells by cleaving sialic acid receptors	None	Inhibitory	Not reported	Not reported	Good
	IFNλ	Phase II (other)	Induces expression of antiviral and inflammatory genes primarily in epithelial cells	None	Inhibitory	No effect	Decreased cell death	Good
	Anti-TRAIL	Preclinical	mAb to TRAIL, blocks interaction between TRAIL and its cognate receptors to inhibit extrinsic apoptosis	None	No effect	No effect	Decreased cell death	Unknown
	A-1155463	Preclinical	Bcl-2 family inhibitor, drives apoptosis of IV infected cells	None	Inhibitory	Not reported	Not reported	Unknown

*Potential therapeutics for human IV infection are summarized. Treatments are separated based on which aspect of IV infection is targeted. Viability of each therapeutic is rated based on data discussed in this review*.

Aside from concerns regarding resistance, the effectiveness of NAIs is limited when delivered over 48 h after symptom onset. Indeed, multiple systematic reviews have concluded that oseltamivir does not reduce IV related hospitalizations, and that there is little evidence of reduction in complications of IV infection (39–42). Although, another meta-analysis did demonstrate that oseltamivir was effective for prevention of influenza at the individual and household levels (43). Use of oseltamivir and other NAIs has demonstrated the need for development of anti-IV drugs that improve treatment effectiveness, particularly when delivered late in the progression of disease, and have a low propensity for driving the emergence of viral resistance.

### Potential IV targeted therapies

The IV surface protein HA binds to host cell receptors to initiate infection. This glycoprotein consists of a globular head and a stem region that are folded within six disulfide bonds, plus several N-glycans that produce a homotrimeric complex structure (44). The majority of IV neutralizing antibodies elicited by vaccination or infection bind to the globular head of HA and recognize homologous strains within a given subtype (45). Antibodies to the HA head neutralize virus infectivity by blocking sialic acid receptor binding either directly, by interacting with the receptor binding site at the tip of the molecule, or indirectly, by projecting over the binding site and rendering it inaccessible (46–48). However, N-linked glycosylation sites on the HA globular head are highly variable across different IV subtypes and some IAV strains can further avoid host antibody responses by acquiring additional N-glycan modifications in the HA head region (49, 50). In contrast, N-linked glycosylation sites in the HA stem region are relatively well conserved among IAV strains. Antibodies to IAV HA stem motifs occur naturally and have activity against a broad range of IAV subtypes, however they are immune-subdominant and are only induced in very low titres during natural infection. Mechanistically, anti-stem antibodies control IAV by inducing antibody-dependent cellular cytotoxicity of infected cells (51–53). Given their potential, several monoclonal antibodies targeting the highly conserved stem region of the HA molecule are being evaluated in clinical trials. In particular, MHAA4549A and MEDI8852 have demonstrated high-affinity binding to 16 IAV HA subtypes and VIS410 has confirmed binding to 7 (54–56) (Table 1). MHAA4549A, MEDI8852, and VIS410 were all shown to be effective in protecting IV infected hosts by inhibiting pulmonary viral load in preclinical animal models (55–59). VIS410 was found to be safe and well tolerated in a phase 1 study and is now under phase 2 investigation (60). MHAA4549A and MEDI8852 were both reported to control viral shedding in humans in phase 2a clinical trials (58, 59). Furthermore, MHAA4549A was reported to lower patient influenza symptom scores and significantly, levels of inflammatory cytokines in serum and nasopharyngeal samples compared to placebo controls (58).

In a clinical trial setting MHAA4549A and MEDI8852 both performed comparably to oseltamivir, yet neither antibody improved oseltamivir effectiveness when used in combination (clinical trials: NCT02293863 and NCT02603952), indicating that these antibodies do not offer better protection than NAIs. However, compared to oseltamivir, which must be given twice daily (61), HA stem antibodies have superior pharmacokinetics, the half-life of MHAA4549A is approximately 3 weeks in humans (58) and MHAA4549A, MEDI8852 and VIS410 all have demonstrated protection against IV induced disease with only one to two doses (55–59). Furthermore, both MHAA4549A and MEDI8852 have been shown to confer protection beyond 48 h post infection, a point at which oseltamivir has lost effectivity in small animal models (55, 56, 58, 59). Excellent pharmacokinetics and a longer therapeutic window make HA stem antibodies strong candidates for treatment of IV infection.

The IV RNA-dependent RNA-polymerase (RdRp), is responsible for transcription and replication of IV's genome and is highly conserved across different strains. It is a heterotrimeric protein containing three virally encoded subunits: PB1, PB2, and PA. PB1 has polymerase activity, PB2 is involved in cap-binding of host cell pre-mRNAs and PA cleaves capped host pre-mRNAs and initiates transcription (62). Cap-snatching by PB2 essential for RNA transcription, PB2 first binds to the 5′-methyl cap of host pre-mRNA which is then cleaved by PA's endonuclease site to produce a capped primer for IV transcription initiation (62). JNJ63623872 (formerly known as VX-787) (Table 1) is a compound that binds to key residues in the PB2 cap binding domain preventing the docking of the natural ligand: 7-methyl GTP. Preclinical *in vivo* and *in vitro* studies have demonstrated that JNJ63623872 has varying degrees of activity against a range of IAV strains, however due to the differences in IAV and IBV PB2 protein JNJ63623872 is ineffective against IBV (63). When directly compared in a mouse model of IAV infection JNJ63623872 was more effective than oseltamivir in controlling IV induced disease severity (64). A placebo-controlled phase IIa study showed JNJ63623872 to be well tolerated and resulted in a 94% reduction in viral shedding and quicker resolution of flu-like symptoms compared to controls (65). However, the dosing regime of JNJ63623872 is similar to oseltamivir and variant strains with reduced susceptibility to JNJ63623872 have been isolated from *in vitro* culture (66), indicating that this therapy in its current form may not supersede NAIs. JNJ63623872 is now in phase II trials alone (NCT02342249) and in combination with oseltamivir (NCT02532283). Interestingly, a phase I trial has been initiated to evaluate the safety and pharmacokinetic interaction of JNJ63623872 with AL-974, a PA inhibitor that is in early-stage development (NCT02888327).

Favipiravir (also known as T705) (Table 1) is a ribonucleotide analog (6-fluoro-3-hydroxy-2-pyrazinecarboxamide) that inhibits viral RdRps. However, the mechanism by which this inhibition occurs is not understood, indeed, even the viral proteins targeted by Favipiravir are not yet defined. *In vitro* studies have revealed that serial passage with increasing concentrations of Favipiravir drives guanosine to adenine nucleotide mutations in IV, essentially resulting in the production of non-viable IV particles (67). Several studies in mice have demonstrated Favipiravir administration up to 72 h post infection with seasonal IAV strains such as H1N1 and avian strains: H5N1 and H7N9 result in a dose-dependent reduction lung viral titres and host mortality (68–71). Favipiravir has been shown to be to have potent inhibitory activity against several RNA viruses *in vitro* and appears especially effective for IVs (72). This acute susceptibility of IV may be due to IV's lack of RNA proofreading enzymes. Furthermore, Favipiravir appears to have an exceptionally high barrier for drug resistance, currently only one mutation (V43I in PB1; obtained in virus-infected cell cultures under selection) was found to confer a slight increase in resistance to Favipiravir (73). Favipiravir is highly promising as a broad acting anti-IV therapy and as such, has been approved for select use in Japan and has completed phase III trials in the USA and Europe.

Along with proteins for replication, assembly and infection, IV genomes also code for a protein which can inhibit the host immune response: non-structural protein (NS1). NS1 is a highly conserved multifunctional protein which inhibits host antiviral responses, particularly, induction of types I and III IFNs (IFNαβ and IFNλ). NS1 antagonism of host immunity varies between IV strains; NS1 can prevent IV-mediated activation of key inflammatory transcription factors such as IFN Regulatory Factor 3 (IRF-3) and nuclear factor kappa-light-chain-enhancer of activated B cells (NF-κB) (74–76). NS1 limits host recognition of IV through the pattern recognition receptor (PRR): retinoic acid inducible gene-I (RIG-I) by sequestering dsRNA (which is a RIG-I agonist) and inhibiting RIG-I ubiquitination and therefore activation (77–83). NS1 is key to viral fitness, strains deficient for NS1 induce markedly higher secretion of antiviral IFNs from cells *in vitro* and are non-pathogenic in mouse models of IV infection (84–87). Thus, the NS1 protein is a suitable target for anti-IV therapeutics. JJ3297 (Table 1) is a second-generation chemical inhibitor of NS1 function that has been shown in an *in vitro* assay to restore levels of IFNαβ-mRNA to those seen when cells were infected with a NS1 deleted mutant (88). While the exact mechanism of action is not understood, JJ3297 mediated inhibition of NS1 absolutely requires the function of cellular RNase L, indicating that an intact interferon system is essential for function of the compound (88). Further development of JJ3297 has resulted in the generation of another compound: A22 and NS1 inhibitors are now being investigated in *in vivo* models of infection (89). Additionally, SP600125, a C-Jun-N-terminal kinase inhibitor reduces the replication of IV *in vitro* and *in vivo* by indirect inhibition of NS1-mediated functions in the early stages of infection (90) and small molecules such as polyphenol and quinoxaline derivatives have also been proposed to inhibit NS1 (91). More study is required to determine if NS1 inhibitors are suitable for clinical use. However, given the direct correlation between host inflammatory response and IV-induced disease severity, use of NS1 inhibitors, particularly late in infection, should be cautiously evaluated.

## Stepping into the storm

Limiting IV replication curbs disease severity not only by decreasing number of virions able to propagate the infection, but also by limiting immune stimulation. All cell types will secrete cytokines and chemokines to varying degrees upon recognition of IV pathogen associated molecular patterns. Cytokines and chemokines drive the recruitment and activation of both innate and adaptive immune cells which, while vital for resolution of infection, can also exacerbate disease through tissue damage. Therefore, at later time points in infection when viral load is already limited, it is more important to control the inflammatory response. Use of interventions which target the host response is an excellent strategy to combat severe IV infection. Host directed therapeutics are unlikely to drive the emergence of resistant strains and their effectivity is not strain specific. However, which immune drives are the most appropriate to target remains an open question. Severe IV infection induces many cytokines; IFNαβ, TNFα, IFNγ, C-X-C motif chemokine (CXCL) 10 (CXCL10), CXCL9, C-C motif ligand (CCL) 2 (CCL2), CCL4, CCL5 and interleukin (IL)−6 (IL-6), IL-2, IL-8, and IL-10 have all be observed to be upregulated during severe IV infection in humans (14, 15, 17, 92, 93). Yet studies in animal models demonstrate that there is yet to be a setting where complete absence of a specific cytokine or its cognate receptor entirely ablates IV induced cytokine storm. As TNFα and IFNαβ correlate well with disease severity in both clinical and experimental IV infection and are potent immunomodulators, known to be upstream of proinflammatory cytokine and chemokine secretion from many cell types, multiple studies have proposed treatment with these cytokines to promote viral clearance, or blockade of these cytokines to minimize host mediated tissue damage 12, 15, 94–101.

TNFα drives the activation of multiple intracellular signaling pathways through the activation of NF-κB (102). In response to IV infection TNFα promotes the secretion of the antiviral cytokine families: type I, II, and III IFNs through upregulating RIG-I and toll-like receptor 3, Myeloid differentiation primary response 88 (MyD88), TIR-domain-containing adapter-inducing interferon-β (TRIF), and IRF7 genes. TNFα drives IV clearance via induction of apoptosis, stimulation of reactive oxygen species and activation of Nicotinamide Adenine Dinucleotide Phosphate Hydrogen (NADPH) oxidases in neutrophils and macrophages, such as NADPH oxidase 2 (NOX2), resulting in the generation of superoxide (103). Yet, TNFα is dispensable for control and clearance of IV, TNF deficient mice exhibited comparable mortality to controls upon H5N1 infection (104). Anti-TNF therapy in a murine H1N1 infection model reduced pulmonary recruitment of inflammatory cells, cytokine production by T cells and the severity of IV induced disease without preventing virus clearance (96). Similarly, treatment of mice lethally infected with H1N1 IAV with etanercept (Table 1), a soluble TNF receptor decoy, significantly reduced inflammatory cell infiltration, production of inflammatory cytokines and downregulated NFκB signaling, yet enhanced host control of virus replication, resulting in a 30% increase in host survival (105).

Interestingly, etanercept is used to treat a range of inflammatory conditions such as Rheumatoid Arthritis (RA). While patients with RA do exhibit an increased risk of IAV infection, treatment with etanercept does not contribute to this. In a retrospective cohort study Blumentals et al. found that etanercept or use of other biologics did not significantly affect the rate of influenza infection or its complications in RA patients (106). Yet whether or not etanercept lowered IV induced disease burden in treated patients compared to controls could not be assessed, as this data was not consistently recorded. Conversely, there is also evidence that TNFα is required for controlling the extent of IV induced immunopathology and tissue injury. In a mouse model of H1N1 infection Damjanovic et al. found that TNF-/- mice exhibited prolonged expression of inflammatory chemokines such as CCL2 leading to an exaggerated immune response and consequent damage to pulmonary epithelial cells (107). Further investigation by DeBerge et al. revealed that it is soluble, and not membrane bound, TNFα that is required to limit the IV induced immune response and tissue damage (108). Therefore, it is unclear if TNFα blockade is a suitable treatment for severe IV induced disease, however given the multiple components of the TNFα signaling system, TNFR1 vs. TNFR2 and the differing activities of membrane bound and soluble TNFα, there is the possibility to specifically inhibit certain aspects of TNFα signaling while not interfering with others.

IFNαβ are the canonical antiviral cytokine family in fact, they were discovered in the context of IV. IFNαβ induces the expression of hundreds of genes, such as MX dynamin like GTPase 1 (Mx1) and interferon induced transmembrane protein 3 (IFITM3) which have direct anti-IV activity. As such, IFNαβ has been periodically suggested as a therapy for IV (94, 97, 100, 101). Prophylactic or very early on treatment with IFNαβ in rhesus macaques, ferrets, guinea pigs and mice experimentally infected with IAV controls virus replication and spread thereby protecting against severe IV induced disease (101, 109–114). However, it appears the therapeutic window is short, later treatment with IFNαβ during infection still controls viral load but exacerbates disease by driving the cytokine storm and TRAIL mediated airway epithelial cell death (101, 109, 115). While there have been no studies directly assessing the effectiveness of IFNαβ blockade during IV infection in humans, IFNαβR deficient mice exhibit a range of susceptibility to IV induced disease depending on the virulence of the infecting IV strain and the genetic background of the mice (86, 115–118), demonstrating that the activity of IFNαβ on host immune response to IV is too complex to extract the immunopathogenic from the protective effects on the host.

Due to the pleiotropic actions of TNFα and IFNαβ direct targeting of these cytokines may not be the most suitable approach. Instead, a general dampening on the immune response may be more effective. Recently, chemical agonism of the sphingosine-1-phosphate (S1P) receptor (S1PR) pathway has been shown to blunt IV induced inflammation. The sphingosine analog: AAL-R (Table 1) agonizes S1P receptors 1, 3, 4, and 5. Treatment of IV infected mice with AAL-R during infection resulted in reduced release of proinflammatory cytokines and chemokines including IFNαβ and inhibited inflammatory cell infiltration and thereby decreased damage to pulmonary tissue. AAL-R treatment did not affect antibody responses and pulmonary viral load was comparable between treatment and control groups, however AAL-R did suppress dendritic cell maturation and inhibited IV specific T-cell responses (119, 120). Although the IV T cell response is dispensable for clearance of IV, it provides the host with herterosubtypic immunity, thus AAL-R is too immunosuppressive to be applied as an anti-IV therapy. But based on the promise of AAL-R, two agonists specific S1P1R: CYM-5442 and RP-002 (Table 1) were tested. Like AAL-R, CYM-5442 and RP-002 significantly reduced cytokine and chemokine responses associated with IV induced lung injury without effecting viral load. Yet, unlike AAL-R, neither CYM-5442 and RP-002 effected dendritic cell and T-cell responses (120, 121). Teijaro et al. proposed that agonism of S1PRs on endothelial cells was responsible for the blunted proinflammatory cytokine levels in the lung (121, 122). However, in follow up studies this group also found that S1P1R agonists act directly on plasmacytoid dendritic cells to block their secretion of IFNα (123, 124). Furthermore, these results defined signaling downstream of MyD88 in multiple cell types to be a key amplifier of IAV induced cytokine storm which could be inhibited by S1P1R agonism. Further characterization of S1PR agonists as IV-therapeutics is ongoing in mouse and ferret models (123).

Cyclooxygenase enzymes (COX) catalyze the conversion of arachidonic acid to prostaglandins, which can modulate the inflammatory response (125). Interestingly, there are two isoforms of COX: the constitutively expressed COX-1 and the inducible COX-2 which have divergent roles in influenza infection. Carey et al. demonstrated that in H3N2 IAV infection COX-2 deficient mice, compared to wild type controls, had lower levels of proinflammatory cytokines (IL-6, TNFα, IL-1β, and IFNγ) and inflammatory cells recruited to the lung during infection, and this correlated to a moderate increase in survival. While in contrast, COX-1 deficient mice in the same study exhibited a higher pulmonary inflammatory burden compared to wild type controls. The cost of this blunted inflammation in COX-2-/- mice was a higher viral burden early in infection, however by day six all three mouse strains had comparably low pulmonary titres of H3N2 IAV (126). In another study, COX-2 deficiency correlated to higher levels of the prostaglandin: PGE_2_ which has an inhibitory effect on proinflammatory cytokine expression, the adaptive immune response and macrophage apoptosis in mice infected with H1N1 (127). Furthermore, COX-2 expression is elevated in autopsy tissue samples from patients infected by H5N1 IAV and induction of proinflammatory cytokines such as IL-6, TNFα, IFNα, and IFNβ by H5N1 in monocyte derived macrophages could be blocked by a COX-2 inhibitor (nimesulide) (128). Thus, there is strong evidence that COX-2 is an upstream driver of IV induced inflammation, however, the specific mechanism of action remains to be determined. In a follow up study, Carey et al. found that treatment of wild type mice with COX-1 inhibitor (SC-560) or a COX-2 inhibitor [celecoxib (Table 1)] prior to and during IAV infection resulted in the same pattern of susceptibility (COX-2 inhibition being protective and COX-1 inhibition being detrimental) yet, neither treatment drastically altered pulmonary cytokine profiles, viral load or inflammatory cell recruitment (129). Furthermore, another *in vivo* study found that celecoxib alone did not protect H5N1 infected mice from mortality, although the authors did observe a protective effect of celecoxib administration when used in combination with zanamvir and mesalazine (a PPARγ agonist, see below) in mice challenged with H5N1 IAV. Significantly, combination treatment was administered post IAV infection. This protection did correlate to a moderate decrease in proinflammatory cytokine concentrations and a modest elevation PGE_2_ in the lung late in infection however, it also correlated to decreased viral loads at this time point which may explain the change in pulmonary cytokine profile (130).

Currently, a phase III clinical trial is running to assess efficacy and safety of celecoxib used in combination with oseltamivir in patients with severe IAV infection (NCT02108366). While this is an exciting development for the use of immunomodulating drugs in the treatment of IV, in high concentrations celecoxib can also inhibit COX-1 (131), which may prove problematic. As demonstrated by Carey et al. COX-1 plays an anti-inflammatory and protective role in IV infection (126). Moreover, treatment with nonselective COX inhibitors such as aspirin and diclofenac confer an increased risk of mortality in animal models of infection and it has been proposed that an increase in aspirin use during the 1918 pandemic contributed to the October death spike (132, 133).

In 2006 Fedson proposed the use of statins to modulate IV induced cytokine storm (134). Statins (Table 1) block cholesterol synthesis by competitively inhibiting the enzyme 3-hydroxy-3-methylglutaryl coenzyme A (HMG-CoA) reductase (135). Commonly employed to reduce the risk of cardiovascular disease by lowering cholesterol levels, statins are inexpensive and widely available, therefore making them an attractive candidate for IV treatment. Statins can inhibit IV induced disease through multiple mechanisms, *in vitro* studies have shown that statins can interfere with viral replication (136, 137), block the induction of proinflammatory cytokines and chemokine such as IL-6 and TNFα and inhibit the activation of key signaling molecules including Signal transducer and activator of transcription 3 (STAT3) (138, 139). Animal studies have shown promise, Haidari et al. demonstrated statin treatment lowered pulmonary viral load and host mortality in murine H3N2 and H1N1 IAV infection models and An et al. demonstrated combination treatment with a statin, a NAI and a fibrate, protected mice from H5N1 mediated mortality (136, 140). In an intriguing study Liu et al. combined statins with another readily available drug: caffeine, and found that combination therapy lowered pulmonary viral load and ameliorated lung damage in H5N1-, H3N2-, and H1N1-infected mice (141). However, other studies conducted in mice have reported little to no effect of statins on IV clearance or cytokine profile (142, 143).

As statins are so widely used in the human population, there is a substantial amount of data on their use in the context of IV infection. Five retrospective studies conducted in four separate countries (Netherlands, United Kingdom, USA and Mexico) reported that to varying degrees, statin treatment associated with reduced IV-related pneumonia and a lower IV induced mortality rate (144–147). In contrast, Fleming et al. and Kwong et al. conducted retrospective studies over a 6 and 10 year periods (respectively) and found no association between statin treatment and decrease IV induced disease burden (148, 149). There are many caveats to these studies, including what other treatments patients were on during the study period and a lack of defined IV specific outcomes. Furthermore, the use of different statins and strains of infecting IVs likely contributes to the varied results. Overall, there is evidence that statins can ameliorate severe IV induced disease, and the availability of this class of drugs certainly makes it an attractive therapeutic option. Further study is required to delineate the specific actions of statins which block viral replication and inhibit over activation of the innate immune response, thereby allowing us to capitalize on these properties. Excitingly, a phase II trial has begun to test the effectivity of atorvastatin in minimizing IV induced disease severity in patients infected with seasonal IV (NCT02056340).

Peroxisome proliferator-activated receptors (PPARs) are nuclear receptors and ligand-activated transcription factors that control a number of target genes upon assembly of a transcriptional complex. PPARs regulate energy balance, including glucose homeostasis, fatty acid oxidation, and lipid metabolism (150). PPAR agonists are commonly used to treat patients with cardiovascular diseases and diabetes mellitus. Drugs which specifically antagonize PPARγ appear to be the most promising as therapeutics for IV. Treatment of mice, prior to and during IAV infection, with PPARγ agonist: pioglitazone (Table 1) was shown to temper recruitment of Ly6C^high^ myeloid cells termed: TNF-α/inducible nitric oxide synthase (iNOS)-producing DCs (tipDCs), although likely these are comparable to what other studies have reported as inflammatory monocytes or exudate macrophages (20, 151, 115). Pioglitazone lowered pulmonary concentrations of chemokines known to attract tipDCs to the lung (CCL2 and CCL7) and this associated with a decrease in IAV induced morbidity and mortality. Importantly, pioglitazone treatment did not alter the rate of IAV clearance from the lung, as was observed when tipDC recruitment was entirely ablated through the genetic deletion of CCR2 (152). In a follow up study, this group also demonstrated that rosiglitazone (another PPARγ agonist) mediated better protection than pioglitazone (or vehicle control) in mice infected with H1N1 IAV (153). Finally, treatment of mice with 15-deoxy-Δ12,14-prostaglandin J2 (15d-PGJ2), 1 day post infection blunted IV induced proinflammatory cytokine secretion in the lung and increased host survival in a PPARγ dependent manner (154). As with statins, PPARγ agonists could be easily employed an adjunct therapy for IV induced disease, however human studies must be performed. Indeed, there is a somewhat surprisingly little amount of data about immunomodulating agents and IV infections. Although imperfect, retrospective studies on patients treated with immunomodulating agents such as IFNαβ for multiple sclerosis or hepatitis C, or any number of anti-inflammatory agents for heart disease may provide informative preliminary data in terms of effectivity and safety.

## Targeting the epithelium

In general, productive IV replication is restricted to airway epithelial cells, as these cells exclusively express proteases required for HA maturation (155). Damage to the respiratory tract in the form of virally induced necrosis, immune mediated apoptosis or other forms of cell death leads to ARDS. Finding a way to directly target the cells which support IV replication is highly desirable in anti IV treatment design. As such, many of the treatments discussed in this review are delivered via inhalation. However, by focusing on features relatively specific to the epithelial cells therapies can directly protect the epithelium during infection or promote healing post viral clearance. For example, Fludase (Table 1), is a recombinant fusion protein consisting of a sialidase catalytic domain derived from *Actinomyces viscosus* fused with the epithelial anchoring domain of human amphiregulin. Fludase is effectively a neuraminidase mimic, it tethers to, and cleaves both α(2,6)-linked and α(2,3)-linked sialic acid receptors, thereby removing IV's entry point into epithelial cells (156). This drug is administered as an inhaled dry powder with microparticles of 5–10 μm in size, enabling the drug to access the upper and central, but not the lower respiratory tract. *In vitro* studies on human airway epithelial cells have shown that Fludase removed approximately 90% of sialic acid receptors within 15 min of treatment and desialylation lasted at least 2 days (157). Serial passage of IAV and IBV under increasing selective pressure of Fludase selected for several mutations in HA (G137R, S136T, S186I) and NA (W438L, L38P) which resulted in IVs with increased receptor binding, coupled with significantly reduced NA on the cell surface. These mutations lead to an attenuated phenotype *in vitro* and no change in virulence in a mouse model of IV infection. Furthermore, the resistance phenotype was unstable and was reversed after withdrawal of Fludase (158).

As it targets the common entry point of IVs Fludase has been shown to be effective at inhibiting a broad range of IAV and IBV strains *in vitro* (159–161). Prophylactic treatment of mice with Fludase inhibited establishment of infection by IAV strains H1N1, H5N1, H7N9 and therefore protected against host mortality. Furthermore, these studies reported that Fludase inhibited IV replication and therefore host mortality when given up to 3 days post infection, albeit with less effectivity than prophylactic treatment (156, 162, 163). Malakhov et al. also demonstrated effectivity of Fludase in a ferret model of H1N1 infection (156). Fludase has begun clinical trials and was generally well tolerated in phase I trial (164). A phase II trial performed over three influenza seasons (2009–2011) in otherwise healthy IV-infected participants demonstrated that Fludase was well tolerated and patients under a multi-dose treatment regime exhibited a significant decrease viral load and viral shedding (165).

While Fludase is a promising anti-IV therapy there are potential pitfalls to broad use. Sialic acid is catabolized by *S. pneumonia*, IV-mediated release of this metabolite is thought to facilitate bacterial colonization and consequent pneumonia (166). In a preclinical study Hedlund et al. demonstrated that Fludase treatment did not alter *S. pneumonia* colonization in an *in vitro* model of a human lung cell line (A549) or in healthy mice. This study also reported that Fludase treatment 24 h post infection with H1N1 or H3N2 strains of IAV protected mice from *S. pneumonia* colonization and therefore morbidity and mortality (167). However, it is important to note that Hedlund et al. administered the secondary bacterial infection 2 days after a single dose of Fludase in IAV infected mice, which, given that airway epithelial cells begin to recover sialylation by 2 days post treatment (157) may be too late to see direct effects of Fludase treatment on bacterial colonization in the context of IV infection. Furthermore, the authors employed a lethal dose of IV, with all vehicle control mice exhibiting highly similar morbidity and mortality regardless of secondary *S. pneumonia* infection. It is therefore unclear whether or not the inoculum of *S. pneumonia* used in this study actually increases disease burden (167). Further studies are required to understand if Fludase alters host susceptibility to secondary bacterial infection.

IFNαβ signal to all cell types in the body and, as discussed, are therefore too inflammatory to be used as anti-influenza therapeutics. However, type III IFNs (IFNλ) (Table 1) are an intriguing alternative. Discovered in 2003, IFNλ are induced during IV infection via the same pathways as IFNαβ and utilize an almost identical signaling cascade to activate transcription of ISGs (168–170). However, IFNλ engages a separate receptor complex with a limited tissue distribution, compared to the ubiquitously expressed IFNαβR. IFNλ receptor expression is predominantly restricted to mucosal surfaces, such as that of the lung, and only select immune cells, primarily neutrophils (86, 169, 171, 172). There is some evidence to suggest IFNλ may be more critical for protection against IV infection than IFNαβ. *In vitro* and *in vivo* analysis has revealed that IFNλ is produced more rapidly and in higher concentrations than IFNαβ by epithelial cells in response to IV infection (101, 170, 172), however this could be attributed to the sensitivity of the assays employed to detect various IFNs. More convincingly, Klinkhammer et al. have recently demonstrated in mice that prophylactic treatment with IFNλ, but not IFNα, confers sustained antiviral protection in the upper airways and blocks IV transmission to uninfected animals (173). In terms of employing IFNλ as in anti-IV therapy, IFNλ treatment consistently administered from 48 to 120 h post infection did not enhance proinflammatory cytokine signaling in the lung but did inhibit IV replication, lowered airway epithelial cell death and consequently promoted host survival (101). Kim et al. reported similar findings and Galani et al. further demonstrated IFNλ signaling to neutrophils also promotes IV clearance (172, 174). Pegylated recombinant IFNλ (PEG-IFNλ) was originally developed to treat Hepatitis C infection, however it was superseded by more specialized treatment options for the disease. Yet during development, PEG-IFNλ passed Phase I and II clinical trials, demonstrating desirable pharmacological properties and a safer drug profile than IFNαβ (175). PEG-IFNλ therefore constitutes a highly promising new broad-spectrum candidate for the treatment IV.

Apoptosis is an important process for resolution of IV infection, not only for elimination of infected cells but also for removing inflammatory cells such as CD8+ T cells, from the pulmonary environment once IV has been cleared. Death-inducing members of the TNF superfamily, including TRAIL and first apoptosis signal (Fas) ligand (FasL) have been shown to induce apoptosis of cells during IV infection (176–180). DNA microarray analysis performed by Kash et al. found that FasL/Fas signaling related genes in the lung are associated with IAV induced mortality in mice (181). Additionally, *ex vivo* assessment of human macrophages has shown that TRAIL expression and secretion is enhanced in severe IV induced disease and human peripheral blood mononuclear cells upregulate TRAIL upon IV infection. Furthermore, IAV infection of a human lung epithelial cell line increases cell susceptibility to TRAIL mediated apoptosis (182, 183). Blocking extrinsic apoptosis by inhibition of Fas/FasL interaction though treatment with a recombinant decoy receptor for FasL or interruption of TRAIL signaling, either by genomic deletion or monoclonal antibody (mAb) blockade (Table 1) during IAV infection can increase the survival rate of mice after IV infection (115, 151, 179, 182–184). Furthermore, mAb blockade of TRAIL signaling protects against secondary bacterial infection (20). Protecting airway epithelial cells from death during IV infection associates with better prognosis. However, it is a fine balance, as mentioned FasL and TRAIL are also used to control inflammatory cells in the lung. Indeed, in severe IAV infection TRAIL deficient mice are more susceptible to IAV induced disease due to accumulation of cytotoxic CD8+ T cells in the lung (180). As yet, blockade of apoptosis in human IV infection has not been assessed.

An alternative approach to entirely blocking apoptosis is to try to target it specifically to infected cells. B-cell lymphoma 2 (Bcl-2) family members such as Bcl-xL, are key regulators of apoptosis and as such Bcl-2 inhibitors have been developed to treat cancer. It was recently proposed that Bcl-2 inhibitors could also be repurposed for antiviral drug development (185). A series of compounds (ABT-737, ABT-263, ABT-199, WEHI-539, A-1331852) have been show to induce premature death of IAV-infected cells at concentrations that were not toxic for non-infected cells *in vitro* (186). Furthermore, Bulanova et al. showed that A-1155463 (Table 1) limited viral spread (186). The authors hypothesize that recognition of IV infection by the cell causes the release of proapoptotic proteins from Bcl-xL to initiate mitochondrial membrane permeabilization, ATP degradation, and caspase-3 activation. Subsequent addition of Bcl-2 inhibitors in low concentrations acts synergistically, further driving apoptosis of IV infected cells. It appears this phenomenon is not specific to IV, as transfection with plasmid DNA elicited similar effects (186, 187). As ABT-199 (as known as Venetoclax) is approved for use in humans for treatment of chronic lymphocytic leukemia, this class of drugs may have potential to be used as anti-IV therapeutics. However, Kakkola et al. did report that ABT-263 treatment of IV-infected mice resulted in an altered pro-inflammatory cytokine profile in the lung and a slightly higher viral load, which associated with decreased host survival, indicating that these treatments may need to be supplemented with other therapeutics which modulate the inflammatory response or promote viral clearance (187).

## Concluding remarks

Globalisation and the continual growth of the world population means that we are living closer together and traveling further distances with greater ease and speed. Emerging strains of IV can transverse the globe in a matter of days. Furthermore, increased demand of fowl and swine products has enlarged the interface between humans and animal reservoirs of IAV, elevating the likelihood of zoonotic transmission. Under these circumstances it is not a case of “if” another IV pandemic emerges but “when.” To combat future IV pandemics we need therapeutics to supplement or replace oseltamivir and other NAIs. Of trials registered on clinicaltrials.gov assessing combination therapies to treat IV (25 results, July 2018), all involve a NAI (primarily oseltamivir) and another therapeutic targeted to IV, with the exception of a single celecoxib/oseltamivir trial (NCT02108366). Combinations of antivirals which inhibit different aspects of IV's replication cycle such as inhibitors for PB and PA (NCT02888327) may have synergistic effects and reduce the likelihood of resistant strains developing. However, trials combining anti-HA stem antibodies and NAIs (NCT02293863 and NCT02603952) have reported no decrease in symptom severity and duration compared to monotherapies. As discussed, severity of IV induced disease is a function of the host immune response, therefore combining antivirals with immunomodulatory drugs will likely prove more effective in treating IV infection. Host directed therapies are less likely to drive drug resistance, are more apt for protecting the delicate epithelium from immune mediated cell death and consequently, may be superior at decreasing disease burden. Repurposing of clinically approved immunomodulators is a simple solution. More trials are needed to assess the feasibility of other immunomodulatory drugs to be used as adjuncts to oseltamivir or other antivirals. Selection of appropriate candidates should be based on *in vivo* models and retrospective studies. Furthermore, taking advantage of inhalers to deliver drugs directly to the site of infection and tailoring therapeutics to epithelial cells, where IV replication occurs will also improve effectivity of treatment while minimizing harmful side effects.

## Author contributions

The author confirms being the sole contributor of this work and approved it for publication.

### Conflict of interest statement

The author declares that the research was conducted in the absence of any commercial or financial relationships that could be construed as a potential conflict of interest.

## References

[1] Stohr K. Influenza–WHO cares. Lancet Infect Dis. (2002) 2:517. 10.1016/S1473-3099(02)00366-3 12206966

[2] Girard MP Cherian T Pervikov Y Kieny MP. A review of vaccine research and development: human acute respiratory infections. Vaccine (2005) 23:5708–24. 10.1016/j.vaccine.2005.07.046 16154667 PMC7130922

[3] Kreijtz JH Fouchier RA Rimmelzwaan GF. Immune responses to influenza virus infection. Virus Res. (2011) 162:19–30. 10.1016/j.virusres.2011.09.022 21963677

[4] Tong S Zhu X Li Y Shi M Zhang J Bourgeois M. New world bats harbor diverse influenza A viruses. PLoS Pathog. (2013) 9:e1003657. 10.1371/journal.ppat.1003657 24130481 PMC3794996

[5] Olsen B Munster VJ Wallensten A Waldenstrom J Osterhaus AD Fouchier RA. Global patterns of influenza a virus in wild birds. Science (2006) 312:384–8. 10.1126/science.1122438 16627734

[6] Wang Z Loh L Kedzierski L Kedzierska K. Avian influenza viruses, inflammation, and CD8(+) T cell immunity. Front Immunol. (2016) 7:60. 10.3389/fimmu.2016.00060 26973644 PMC4771736

[7] Robinson JL Lee BE Patel J Bastien N Grimsrud K Seal RF. Swine influenza (H3N2) infection in a child and possible community transmission, Canada. Emerg Infect Dis. (2007) 13:1865–70. 10.3201/eid1312.070615 18258037 PMC2876760

[8] Kucharski AJ Lessler J Read JM Zhu H Jiang CQ Guan Y. Estimating the life course of influenza A(H3N2) antibody responses from cross-sectional data. PLoS Biol. (2015) 13:e1002082. 10.1371/journal.pbio.1002082 25734701 PMC4348415

[9] Hensley SE Das SR Bailey AL Schmidt LM Hickman HD Jayaraman A. Hemagglutinin receptor binding avidity drives influenza A virus antigenic drift. Science (2009) 326:734–6. 10.1126/science.1178258 19900932 PMC2784927

[10] Taubenberger JK. The origin and virulence of the 1918 “Spanish” influenza virus. Proc Am Philos Soc. (2006) 150:86–112. 17526158 PMC2720273

[11] Short KR Kroeze E Fouchier RM Kuiken T. Pathogenesis of influenza-induced acute respiratory distress syndrome. Lancet Infect Dis. (2014). 14:57–69. 10.1016/S1473-3099(13)70286-X 24239327

[12] Hayden FG Fritz R Lobo MC Alvord W Strober W Straus SE. Local and systemic cytokine responses during experimental human influenza A virus infection. Relation to symptom formation and host defense. J Clin Invest. (1998) 101:643–9. 10.1172/JCI1355 9449698 PMC508608

[13] Kaiser L Fritz RS Straus SE Gubareva L Hayden FG. Symptom pathogenesis during acute influenza: interleukin-6 and other cytokine responses. J Med Virol. (2001) 64:262–8. 10.1002/jmv.1045 11424113

[14] Peiris JS Yu WC Leung CW Cheung CY Ng WF Nicholls JM. Re-emergence of fatal human influenza A subtype H5N1 disease. Lancet (2004) 363:617–9. 10.1016/S0140-6736(04)15595-5 14987888 PMC7112424

[15] De Jong MD Simmons CP Thanh TT Hien VM Smith GJ Chau TN. Fatal outcome of human influenza A (H5N1) is associated with high viral load and hypercytokinemia. Nat Med. (2006) 12:1203–7. 10.1038/nm1477 16964257 PMC4333202

[16] Louie JK Acosta M Winter K Jean C Gavali S Schechter R. Factors associated with death or hospitalization due to pandemic 2009 influenza A(H1N1) infection in California. JAMA (2009) 302:1896–902. 10.1001/jama.2009.1583 19887665

[17] Arankalle VA Lole KS Arya RP Tripathy AS Ramdasi AY Chadha MS. Role of host immune response and viral load in the differential outcome of pandemic H1N1 (2009) influenza virus infection in Indian patients. PLoS ONE (2010) 5:e13099. 10.1371/journal.pone.0013099 20957032 PMC2948498

[18] Mccullers JA. The co-pathogenesis of influenza viruses with bacteria in the lung. Nat Rev Microbiol. (2014) 12:252–62. 10.1038/nrmicro3231 24590244

[19] Shahangian A Chow EK Tian X Kang JR Ghaffari A Liu SY. Type I IFNs mediate development of postinfluenza bacterial pneumonia in mice. J Clin Invest. (2009) 119:1910–20. 10.1172/JCI35412 19487810 PMC2701856

[20] Ellis GT Davidson S Crotta S Branzk N Papayannopoulos V Wack A. TRAIL+ monocytes and monocyte-related cells cause lung damage and thereby increase susceptibility to influenza-Streptococcus pneumoniae coinfection. EMBO Rep. (2015) 16:1203–18. 10.15252/embr.201540473 26265006 PMC4576987

[21] Rajao DS Perez DR. Universal vaccines and vaccine platforms to protect against influenza viruses in humans and agriculture. Front Microbiol. (2018) 9:123. 10.3389/fmicb.2018.00123 29467737 PMC5808216

[22] Shtyrya YA Mochalova LV Bovin NV. Influenza virus neuraminidase: structure and function. Acta Naturae (2009) 1:26–32. 22649600 PMC3347517

[23] Cohen M Zhang XQ Senaati HP Chen HW Varki NM Schooley RT. Influenza A penetrates host mucus by cleaving sialic acids with neuraminidase. Virol J. (2013) 10:321. 10.1186/1743-422X-10-321 24261589 PMC3842836

[24] Colman PM Hoyne PA Lawrence MC. Sequence and structure alignment of paramyxovirus hemagglutinin-neuraminidase with influenza virus neuraminidase. J Virol. (1993) 67:2972–80. 8497041 10.1128/jvi.67.6.2972-2980.1993 PMC237633

[25] Kamali A Holodniy M. Influenza treatment and prophylaxis with neuraminidase inhibitors: a review. Infect Drug Resist. (2013) 6:187–98. 10.2147/IDR.S36601 24277988 PMC3838482

[26] Ison MG. Antiviral treatments. Clin Chest Med. (2017) 38:139–53. 10.1016/j.ccm.2016.11.008 28159156 PMC7131036

[27] Mendel DB Roberts NA. *In-vitro* and *in-vivo* efficacy of influenza neuraminidase inhibitors. Curr Opin Infect Dis. (1998) 11:727–32. 10.1097/00001432-199812000-00013 17035750

[28] Dharan NJ Gubareva LV Meyer JJ Okomo-Adhiambo M Mcclinton RC Marshall SA. Infections with oseltamivir-resistant influenza A(H1N1) virus in the United States. JAMA (2009) 301:1034–41. 10.1001/jama.2009.294 19255110

[29] Hurt AC Ernest J Deng YM Iannello P Besselaar TG Birch C. Emergence and spread of oseltamivir-resistant A(H1N1) influenza viruses in Oceania, South East Asia and South Africa. Antiviral Res. (2009a) 83:90–3. 10.1016/j.antiviral.2009.03.003 19501261

[30] Hurt AC Holien JK Parker MW Barr IG. Oseltamivir resistance and the H274Y neuraminidase mutation in seasonal, pandemic and highly pathogenic influenza viruses. Drugs (2009b) 69:2523–31. 10.2165/11531450-000000000-00000 19943705

[31] Meijer A Lackenby A Hungnes O Lina B Van-Der-Werf S Schweiger B. Oseltamivir-resistant influenza virus A (H1N1), Europe, 2007-08 season. Emerg Infect Dis. (2009) 15:552–60. 10.3201/eid1504.181280 19331731 PMC2671453

[32] Collins PJ Haire LF Lin YP Liu J Russell RJ Walker PA. Crystal structures of oseltamivir-resistant influenza virus neuraminidase mutants. Nature (2008) 453:1258–61. 10.1038/nature06956 18480754

[33] Malaisree M Rungrotmongkol T Nunthaboot N Aruksakunwong O Intharathep P Decha P. Source of oseltamivir resistance in avian influenza H5N1 virus with the H274Y mutation. Amino Acids (2009) 37:725–32. 10.1007/s00726-008-0201-z 19002747

[34] Wang MZ Tai CY Mendel DB. Mechanism by which mutations at his274 alter sensitivity of influenza a virus n1 neuraminidase to oseltamivir carboxylate and zanamivir. Antimicrob Agents Chemother. (2002) 46:3809–16. 10.1128/AAC.46.12.3809-3816.2002 12435681 PMC132783

[35] Moscona A. Global transmission of oseltamivir-resistant influenza. N Engl J Med. (2009) 360:953–6. 10.1056/NEJMp0900648 19258250

[36] Weinstock DM Zuccotti G. The evolution of influenza resistance and treatment. JAMA (2009) 301:1066–9. 10.1001/jama.2009.324 19255112

[37] Hurt AC Hardie K Wilson NJ Deng YM Osbourn M Gehrig N. Community transmission of oseltamivir-resistant A(H1N1)pdm09 influenza. N Engl J Med. (2011) 365:2541–2. 10.1056/NEJMc1111078 22204735

[38] Wang D Sleeman K Huang W Nguyen HT Levine M Cheng Y. Neuraminidase inhibitor susceptibility testing of influenza type B viruses in China during 2010 and 2011 identifies viruses with reduced susceptibility to oseltamivir and zanamivir. Antiviral Res. (2013) 97:240–4. 10.1016/j.antiviral.2012.12.013 23267831

[39] Ebell MH Call M Shinholser J. Effectiveness of oseltamivir in adults: a meta-analysis of published and unpublished clinical trials. Fam Pract. (2013) 30:125–33. 10.1093/fampra/cms059 22997224

[40] Michiels B Van Puyenbroeck K Verhoeven V Vermeire E Coenen S. The value of neuraminidase inhibitors for the prevention and treatment of seasonal influenza: a systematic review of systematic reviews. PLoS ONE (2013) 8:e60348. 10.1371/journal.pone.0060348 23565231 PMC3614893

[41] Jefferson T Jones M Doshi P Spencer EA Onakpoya I Heneghan CJ. Oseltamivir for influenza in adults and children: systematic review of clinical study reports and summary of regulatory comments. BMJ (2014) 348:g2545. 10.1136/bmj.g2545 24811411 PMC3981975

[42] Jefferson T Jones MA Doshi P Del Mar CB Hama R Thompson MJ Neuraminidase inhibitors for preventing and treating influenza in healthy adults and children. Cochrane Database Syst Rev. (2014) CD008965 10.1002/14651858.CD008965.pub3 22258996

[43] Okoli GN Otete HE Beck CR Nguyen-Van-Tam JS. Use of neuraminidase inhibitors for rapid containment of influenza: a systematic review and meta-analysis of individual and household transmission studies. PLoS ONE (2014) 9:e113633. 10.1371/journal.pone.0113633 25490762 PMC4260958

[44] Skehel JJ Wiley DC. Receptor binding and membrane fusion in virus entry: the influenza hemagglutinin. Annu Rev Biochem. (2000) 69:531–69. 10.1146/annurev.biochem.69.1.531 10966468

[45] Russell RJ Kerry PS Stevens DJ Steinhauer DA Martin SR Gamblin SJ. Structure of influenza hemagglutinin in complex with an inhibitor of membrane fusion. Proc Natl Acad Sci USA. (2008) 105:17736–41. 10.1073/pnas.0807142105 19004788 PMC2584702

[46] Fleury D Barrere B Bizebard T Daniels RS Skehel JJ Knossow M. A complex of influenza hemagglutinin with a neutralizing antibody that binds outside the virus receptor binding site. Nat Struct Biol. (1999) 6:530–4. 10.1038/9299 10360354

[47] Knossow M Skehel JJ. Variation and infectivity neutralization in influenza. Immunology (2006) 119:1–7. 10.1111/j.1365-2567.2006.02421.x 16925526 PMC1782343

[48] Xiong X Corti D Liu J Pinna D Foglierini M Calder LJ. Structures of complexes formed by H5 influenza hemagglutinin with a potent broadly neutralizing human monoclonal antibody. Proc Natl Acad Sci USA. (2015) 112:9430–5. 10.1073/pnas.1510816112 26170284 PMC4522749

[49] Das SR Puigbo P Hensley SE Hurt DE Bennink JR Yewdell JW. Glycosylation focuses sequence variation in the influenza A virus H1 hemagglutinin globular domain. PLoS Pathog. (2010) 6:e1001211. 10.1371/journal.ppat.1001211 21124818 PMC2991263

[50] Medina RA Stertz S Manicassamy B Zimmermann P Sun X Albrecht R A. Glycosylations in the globular head of the hemagglutinin protein modulate the virulence and antigenic properties of the H1N1 influenza viruses. Sci Transl Med. (2013) 5:187ra170. 10.1126/scitranslmed.3005996 23720581 PMC3940933

[51] Corti D Voss J Gamblin SJ Codoni G Macagno A Jarrossay D. A neutralizing antibody selected from plasma cells that binds to group 1 and group 2 influenza A hemagglutinins. Science (2011) 333:850–6. 10.1126/science.1205669 21798894

[52] Dilillo DJ Tan GS Palese P Ravetch JV. Broadly neutralizing hemagglutinin stalk-specific antibodies require FcgammaR interactions for protection against influenza virus *in vivo*. Nat Med. (2014) 20:143–51. 10.1038/nm.3443 24412922 PMC3966466

[53] Dilillo DJ Palese P Wilson PC Ravetch JV. Broadly neutralizing anti-influenza antibodies require Fc receptor engagement for *in vivo* protection. J Clin Invest. (2016) 126:605–10. 10.1172/JCI84428 26731473 PMC4731186

[54] Tharakaraman K Subramanian V Cain D Sasisekharan V Sasisekharan R. Broadly neutralizing influenza hemagglutinin stem-specific antibody CR8020 targets residues that are prone to escape due to host selection pressure. Cell Host Microbe (2014) 15:644–51. 10.1016/j.chom.2014.04.009 24832457 PMC4258880

[55] Gupta P Kamath AV Park S Chiu H Lutman J Maia M. Preclinical pharmacokinetics of MHAA4549A, a human monoclonal antibody to influenza A virus, and the prediction of its efficacious clinical dose for the treatment of patients hospitalized with influenza A. MAbs (2016) 8:991–7. 10.1080/19420862.2016.1167294 27031797 PMC5037985

[56] Kallewaard NL Corti D Collins PJ Neu U Mcauliffe JM Benjamin E. Structure and function analysis of an antibody recognizing all influenza A subtypes. Cell (2016) 166:596–608. 10.1016/j.cell.2016.05.073 27453466 PMC4967455

[57] Baranovich T Jones JC Russier M Vogel P Szretter KJ Sloan SE. The hemagglutinin stem-binding monoclonal antibody VIS410 controls influenza virus-induced acute respiratory distress syndrome. Antimicrob Agents Chemother. (2016) 60:2118–31. 10.1128/AAC.02457-15 26787699 PMC4808199

[58] Mcbride JM Lim JJ Burgess T Deng R Derby MA Maia M. Phase 2 randomized trial of the safety and efficacy of MHAA4549A, a broadly neutralizing monoclonal antibody, in a human influenza A virus challenge model. Antimicrob Agents Chemother. (2017) 61:e01154–17. 10.1128/AAC.01154-17 28807912 PMC5655070

[59] Omar Ali TT Andrew C Nyborg KM Jensen FD Raburn M A phase 2a study to evaluate the safety of MEDI8852 in outpatient adults with acute, uncomplicated influenza A. Open Forum Infect Dis. (2017) 4:S519 10.1093/ofid/ofx163.1352

[60] Wollacott AM Boni MF Szretter KJ Sloan SE Yousofshahi M Viswanathan K. Safety and upper respiratory pharmacokinetics of the hemagglutinin stalk-binding antibody VIS410 support treatment and prophylaxis based on population modeling of seasonal influenza A outbreaks. EBioMedicine (2016) 5:147–55. 10.1016/j.ebiom.2016.02.021 27077121 PMC4816807

[61] Rayner CR Bulik CC Kamal MA Reynolds DK Toovey S Hammel JP. Pharmacokinetic-pharmacodynamic determinants of oseltamivir efficacy using data from phase 2 inoculation studies. Antimicrob Agents Chemother. (2013) 57:3478–87. 10.1128/AAC.02440-12 23669386 PMC3719781

[62] Martin-Benito J Ortin J. Influenza virus transcription and replication. Adv Virus Res. (2013) 87:113–37. 10.1016/B978-0-12-407698-3.00004-1 23809922

[63] Fu Y Gaelings L Soderholm S Belanov S Nandania J Nyman TA. JNJ872 inhibits influenza A virus replication without altering cellular antiviral responses. Antiviral Res. (2016) 133:23–31. 10.1016/j.antiviral.2016.07.008 27451344

[64] Smee DF Barnard DL Jones SM. Activities of JNJ63623872 and oseltamivir against influenza A H1N1pdm and H3N2 virus infections in mice. Antiviral Res. (2016) 136:45–50. 10.1016/j.antiviral.2016.10.009 27771390

[65] VRTX-GEN VX-787 Showed Significant Antiviral Activity and Reduced the Severity and Duration of Influenza Symptoms in Phase 2 Challenge Study. Boston, MA (2016).

[66] Clark MP Ledeboer MW Davies I Byrn RA Jones SM Perola E. Discovery of a novel, first-in-class, orally bioavailable azaindole inhibitor (VX-787) of influenza PB2. J Med Chem. (2014) 57:6668–78. 10.1021/jm5007275 25019388

[67] Baranovich T Wong SS Armstrong J Marjuki H Webby RJ Webster RG. T-705 (favipiravir) induces lethal mutagenesis in influenza A H1N1 viruses *in vitro*. J Virol. (2013) 87:3741–51. 10.1128/JVI.02346-12 23325689 PMC3624194

[68] Furuta Y Takahashi K Fukuda Y Kuno M Kamiyama T Kozaki K. *In vitro* and *in vivo* activities of anti-influenza virus compound T-705. Antimicrob Agents Chemother. (2002) 46:977–81. 10.1128/AAC.46.4.977-981.2002 11897578 PMC127093

[69] Sidwell RW Barnard DL Day CW Smee DF Bailey KW Wong MH. Efficacy of orally administered T-705 on lethal avian influenza A (H5N1) virus infections in mice. Antimicrob Agents Chemother. (2007) 51:845–51. 10.1128/AAC.01051-06 17194832 PMC1803113

[70] Kiso M Takahashi K Sakai-Tagawa Y Shinya K Sakabe S Le QM. T-705 (favipiravir) activity against lethal H5N1 influenza A viruses. Proc Natl Acad Sci USA. (2010) 107:882–7. 10.1073/pnas.0909603107 20080770 PMC2818889

[71] Furuta Y Gowen BB Takahashi K Shiraki K Smee DF Barnard DL. Favipiravir (T-705), a novel viral RNA polymerase inhibitor. Antiviral Res. (2013) 100:446–54. 10.1016/j.antiviral.2013.09.015 24084488 PMC3880838

[72] Furuta Y Takahashi K Kuno-Maekawa M Sangawa H Uehara S Kozaki K. Mechanism of action of T-705 against influenza virus. Antimicrob Agents Chemother. (2005) 49:981–6. 10.1128/AAC.49.3.981-986.2005 15728892 PMC549233

[73] Cheung PP Watson SJ Choy KT Fun Sia S Wong DD Poon LL. Generation and characterization of influenza A viruses with altered polymerase fidelity. Nat Commun. (2014) 5:4794. 10.1038/ncomms5794 25183443 PMC4155405

[74] Talon J Horvath CM Polley R Basler CF Muster T Palese P. Activation of interferon regulatory factor 3 is inhibited by the influenza A virus NS1 protein. J Virol. (2000) 74:7989–96. 10.1128/JVI.74.17.7989-7996.2000 10933707 PMC112330

[75] Wang X Li M Zheng H Muster T Palese P Beg AA. Influenza A virus NS1 protein prevents activation of NF-kappaB and induction of alpha/beta interferon. J Virol. (2000) 74:11566–73. 10.1128/JVI.74.24.11566-11573.2000 11090154 PMC112437

[76] Ludwig S Wang X Ehrhardt C Zheng H Donelan N Planz O. The influenza A virus NS1 protein inhibits activation of Jun N-terminal kinase and AP-1 transcription factors. J Virol. (2002) 76:11166–71. 10.1128/JVI.76.21.11166-11171.2002 12368362 PMC136597

[77] Donelan NR Basler CF Garcia-Sastre A. A recombinant influenza A virus expressing an RNA-binding-defective NS1 protein induces high levels of beta interferon and is attenuated in mice. J Virol. (2003) 77:13257–66. 10.1128/JVI.77.24.13257-13266.2003 14645582 PMC296096

[78] Pichlmair A Schulz O Tan CP Naslund TI Liljestrom P Weber F. RIG-I-mediated antiviral responses to single-stranded RNA bearing 5'-phosphates. Science (2006) 314:997–1001. 10.1126/science.1132998 17038589

[79] Gack MU Shin YC Joo CH Urano T Liang C Sun L. TRIM25 RING-finger E3 ubiquitin ligase is essential for RIG-I-mediated antiviral activity. Nature (2007) 446:916–20. 10.1038/nature05732 17392790

[80] Guo Z Chen LM Zeng H Gomez JA Plowden J Fujita T. NS1 protein of influenza A virus inhibits the function of intracytoplasmic pathogen sensor, RIG-I. Am J Respir Cell Mol Biol. (2007) 36:263–9. 10.1165/rcmb.2006-0283RC 17053203

[81] Mibayashi M Martinez-Sobrido L Loo YM Cardenas WB Gale M Jr Garcia-Sastre A. Inhibition of retinoic acid-inducible gene I-mediated induction of beta interferon by the NS1 protein of influenza A virus. J Virol. (2007) 81:514–24. 10.1128/JVI.01265-06 17079289 PMC1797471

[82] Opitz B Rejaibi A Dauber B Eckhard J Vinzing M Schmeck B. IFNbeta induction by influenza A virus is mediated by RIG-I which is regulated by the viral NS1 protein. Cell Microbiol. (2007) 9:930–8. 10.1111/j.1462-5822.2006.00841.x 17140406

[83] Steidle S Martinez-Sobrido L Mordstein M Lienenklaus S Garcia-Sastre A Staheli P. Glycine 184 in nonstructural protein NS1 determines the virulence of influenza A virus strain PR8 without affecting the host interferon response. J Virol. (2010) 84:12761–70. 10.1128/JVI.00701-10 20926573 PMC3004359

[84] Garcia-Sastre A Egorov A Matassov D Brandt S Levy DE Durbin JE. Influenza A virus lacking the NS1 gene replicates in interferon-deficient systems. Virology (1998) 252:324–30. 10.1006/viro.1998.9508 9878611

[85] Diebold SS Montoya M Unger H Alexopoulou L Roy P Haswell LE. Viral infection switches non-plasmacytoid dendritic cells into high interferon producers. Nature (2003) 424:324–8. 10.1038/nature01783 12819664

[86] Mordstein M Kochs G Dumoutier L Renauld JC Paludan SR Klucher K Interferon-lambda contributes to innate immunity of mice against influenza A virus but not against hepatotropic viruses. PLoS Pathog. (2008) 4:e1000151 10.1371/journal.ppat.1000151 18787692 PMC2522277

[87] Kallfass C Lienenklaus S Weiss S Staeheli P Visualizing the beta interferon response in mice during infection with influenza A viruses expressing or lacking nonstructural protein 1. J Virol. (2013) 87:6925–30. 10.1128/JVI.00283-13 23576514 PMC3676098

[88] Walkiewicz MP Basu D Jablonski JJ Geysen HM Engel DA. Novel inhibitor of influenza non-structural protein 1 blocks multi-cycle replication in an RNase L-dependent manner. J Gen Virol. (2011) 92:60–70. 10.1099/vir.0.025015-0 20881091 PMC3052532

[89] Jablonski JJ Basu D Engel DA Geysen HM. Design, synthesis, and evaluation of novel small molecule inhibitors of the influenza virus protein NS1. Bioorg Med Chem. (2012) 20:487–97. 10.1016/j.bmc.2011.10.026 22099257 PMC4373408

[90] Zhang S Tian H Cui J Xiao J Wang M Hu Y. The c-Jun N-terminal kinase (JNK) is involved in H5N1 influenza A virus RNA and protein synthesis. Arch Virol. (2016) 161:345–51. 10.1007/s00705-015-2668-8 26559961

[91] Monod A Swale C Tarus B Tissot A Delmas B Ruigrok RW. Learning from structure-based drug design and new antivirals targeting the ribonucleoprotein complex for the treatment of influenza. Expert Opin Drug Discov. (2015) 10:345–71. 10.1517/17460441.2015.1019859 25792362

[92] Beigel JH Farrar J Han AM Hayden FG Hyer R De Jong MD. Avian influenza A (H5N1) infection in humans. N Engl J Med. (2005) 353:1374–85. 10.1056/NEJMra052211 16192482

[93] Lichtner M Mastroianni CM Rossi R Russo G Belvisi V Marocco R. Severe and persistent depletion of circulating plasmacytoid dendritic cells in patients with 2009 pandemic H1N1 infection. PLoS ONE (2011) 6:e19872. 10.1371/journal.pone.0019872 21625541 PMC3098245

[94] Finter NB Chapman S Dowd P Johnston JM Manna V Sarantis N. The use of interferon-alpha in virus infections. Drugs (1991) 42:749–65. 10.2165/00003495-199142050-00003 1723372 PMC7100942

[95] Fritz RS Hayden FG Calfee DP Cass LM Peng AW Alvord WG. Nasal cytokine and chemokine responses in experimental influenza A virus infection: results of a placebo-controlled trial of intravenous zanamivir treatment. J Infect Dis. (1999) 180:586–93. 10.1086/314938 10438343

[96] Hussell T Pennycook A Openshaw PJ. Inhibition of tumor necrosis factor reduces the severity of virus-specific lung immunopathology. Eur J Immunol. (2001) 31:2566–573. 10.1002/1521-4141(200109)31:9<2566::AID-IMMU2566>3.0.CO;2-L 11536154

[97] Mckinlay MA. Recent advances in the treatment of rhinovirus infections. Curr Opin Pharmacol. (2001) 1:477–81. 10.1016/S1471-4892(01)00083-2 11764773

[98] Kim B Ahn KK Ha Y Lee YH Kim D Lim JH. Association of tumor necrosis factor-alpha with fever and pulmonary lesion score in pigs experimentally infected with swine influenza virus subtype H1N2. J Vet Med Sci. (2009) 71:611–6. 10.1292/jvms.71.611 19498287

[99] Shale M Czub M Kaplan GG Panaccione R Ghosh S. Anti-tumor necrosis factor therapy and influenza: keeping it in perspective. Therap Adv Gastroenterol. (2010) 3:173–7. 10.1177/1756283X10366368 21180599 PMC3002575

[100] Wang Z Zhang A Wan Y Liu X Qiu C Xi X. Early hypercytokinemia is associated with interferon-induced transmembrane protein-3 dysfunction and predictive of fatal H7N9 infection. Proc Natl Acad Sci USA. (2014) 111:769–74. 10.1073/pnas.1321748111 24367104 PMC3896201

[101] Davidson S Mccabe TM Crotta S Gad HH Hessel EM Beinke S. IFNlambda is a potent anti-influenza therapeutic without the inflammatory side effects of IFNalpha treatment. EMBO Mol Med. (2016) 8:1099–112. 10.15252/emmm.201606413 27520969 PMC5009813

[102] Sethu S Melendez AJ. New developments on the TNFalpha-mediated signalling pathways. Biosci Rep. (2011) 31:63–76. 10.1042/BSR20100040 20964626

[103] Matikainen S Siren J Tissari J Veckman V Pirhonen J Severa M. Tumor necrosis factor alpha enhances influenza A virus-induced expression of antiviral cytokines by activating RIG-I gene expression. J Virol. (2006) 80:3515–22. 10.1128/JVI.80.7.3515-3522.2006 16537619 PMC1440408

[104] Szretter KJ Gangappa S Lu X Smith C Shieh WJ Zaki SR. Role of host cytokine responses in the pathogenesis of avian H5N1 influenza viruses in mice. J Virol. (2007) 81:2736–44. 10.1128/JVI.02336-06 17182684 PMC1866007

[105] Shi X Zhou W Huang H Zhu H Zhou P Zhu H. Inhibition of the inflammatory cytokine tumor necrosis factor-alpha with etanercept provides protection against lethal H1N1 influenza infection in mice. Crit Care (2013) 17:R301. 10.1186/cc13171 24373231 PMC4057515

[106] Blumentals WA Arreglado A Napalkov P Toovey S. Rheumatoid arthritis and the incidence of influenza and influenza-related complications: a retrospective cohort study. BMC Musculoskelet Disord. (2012) 13:158. 10.1186/1471-2474-13-158 22925480 PMC3495205

[107] Damjanovic D Divangahi M Kugathasan K Small CL Zganiacz A Brown EG. Negative regulation of lung inflammation and immunopathology by TNF-alpha during acute influenza infection. Am J Pathol. (2011) 179:2963–76. 10.1016/j.ajpath.2011.09.003 22001698 PMC3260802

[108] Deberge MP Ely KH Enelow RI Soluble, but not transmembrane, TNF-alpha is required during influenza infection to limit the magnitude of immune responses and the extent of immunopathology. J Immunol. (2014) 192:5839–51. 10.4049/jimmunol.1302729 24790150 PMC4048787

[109] Beilharz MW Cummins JM Bennett AL. Protection from lethal influenza virus challenge by oral type 1 interferon. Biochem Biophys Res Commun. (2007) 355:740–4. 10.1016/j.bbrc.2007.02.019 17316562

[110] Tumpey TM Szretter KJ Van Hoeven N Katz JM Kochs G Haller O. The Mx1 gene protects mice against the pandemic 1918 and highly lethal human H5N1 influenza viruses. J Virol. (2007) 81:10818–21. 10.1128/JVI.01116-07 17652381 PMC2045488

[111] Kugel D Kochs G Obojes K Roth J Kobinger GP Kobasa D. Intranasal administration of alpha interferon reduces seasonal influenza A virus morbidity in ferrets. J Virol. (2009) 83:3843–51. 10.1128/JVI.02453-08 19193792 PMC2663257

[112] Van Hoeven N Belser JA Szretter KJ Zeng H Staeheli P Swayne DE. Pathogenesis of 1918 pandemic and H5N1 influenza virus infections in a guinea pig model: antiviral potential of exogenous alpha interferon to reduce virus shedding. J Virol. (2009) 83:2851–61. 10.1128/JVI.02174-08 19144714 PMC2655560

[113] Matzinger SR Carroll TD Fritts L Mcchesney MB Miller CJ. Exogenous IFN-alpha administration reduces influenza A virus replication in the lower respiratory tract of rhesus macaques. PLoS ONE (2011) 6:e29255. 10.1371/journal.pone.0029255 22220209 PMC3248419

[114] Cilloniz C Pantin-Jackwood MJ Ni C Carter VS Korth MJ Swayne DE. Molecular signatures associated with Mx1-mediated resistance to highly pathogenic influenza virus infection: mechanisms of survival. J Virol. (2012) 86:2437–46. 10.1128/JVI.06156-11 22190720 PMC3302269

[115] Davidson S Crotta S Mccabe TM Wack A. Pathogenic potential of interferon alphabeta in acute influenza infection. Nat Commun. (2014) 5:3864. 10.1038/ncomms4864 24844667 PMC4033792

[116] Price GE Gaszewska-Mastarlarz A Moskophidis D. The role of alpha/beta and gamma interferons in development of immunity to influenza A virus in mice. J Virol. (2000) 74:3996–4003. 10.1128/JVI.74.9.3996-4003.2000 10756011 PMC111913

[117] Koerner I Kochs G Kalinke U Weiss S Staeheli P. Protective role of beta interferon in host defense against influenza A virus. J Virol. (2007) 81:2025–30. 10.1128/JVI.01718-06 17151098 PMC1797552

[118] Szretter KJ Gangappa S Belser JA Zeng H Chen H Matsuoka Y. Early control of H5N1 influenza virus replication by the type I interferon response in mice. J Virol. (2009) 83:5825–34. 10.1128/JVI.02144-08 19297490 PMC2681972

[119] Marsolais D Rosen H. Chemical modulators of sphingosine-1-phosphate receptors as barrier-oriented therapeutic molecules. Nat Rev Drug Discov. (2009) 8:297–307. 10.1038/nrd2356 19300460 PMC4455967

[120] Walsh KB Teijaro JR Wilker PR Jatzek A Fremgen DM Das SC. Suppression of cytokine storm with a sphingosine analog provides protection against pathogenic influenza virus. Proc Natl Acad Sci USA. (2011) 108:12018–23. 10.1073/pnas.1107024108 21715659 PMC3142000

[121] Teijaro JR Walsh KB Cahalan S Fremgen DM Roberts E Scott F. Endothelial cells are central orchestrators of cytokine amplification during influenza virus infection. Cell (2011) 146:980–91. 10.1016/j.cell.2011.08.015 21925319 PMC3176439

[122] Matheu MP Teijaro JR Walsh KB Greenberg ML Marsolais D Parker I. Three phases of CD8 T cell response in the lung following H1N1 influenza infection and sphingosine 1 phosphate agonist therapy. PLoS ONE (2013) 8:e58033. 10.1371/journal.pone.0058033 23533579 PMC3606384

[123] Teijaro JR Walsh KB Rice S Rosen H Oldstone MB. Mapping the innate signaling cascade essential for cytokine storm during influenza virus infection. Proc Natl Acad Sci USA. (2014) 111:3799–804. 10.1073/pnas.1400593111 24572573 PMC3956176

[124] Teijaro JR Studer S Leaf N Kiosses WB Nguyen N Matsuki K. S1PR1-mediated IFNAR1 degradation modulates plasmacytoid dendritic cell interferon-alpha autoamplification. Proc Natl Acad Sci USA. (2016) 113:1351–6. 10.1073/pnas.1525356113 26787880 PMC4747766

[125] Ricciotti E Fitzgerald GA. Prostaglandins and inflammation. Arterioscler Thromb Vasc Biol. (2011) 31:986–1000. 10.1161/ATVBAHA.110.207449 21508345 PMC3081099

[126] Carey MA Bradbury JA Seubert JM Langenbach R Zeldin DC Germolec DR. Contrasting effects of cyclooxygenase-1 (COX-1) and COX-2 deficiency on the host response to influenza A viral infection. J Immunol. (2005) 175:6878–84. 10.4049/jimmunol.175.10.6878 16272346

[127] Coulombe F Jaworska J Verway M Tzelepis F Massoud A Gillard J. Targeted prostaglandin E2 inhibition enhances antiviral immunity through induction of type I interferon and apoptosis in macrophages. Immunity (2014) 40:554–68. 10.1016/j.immuni.2014.02.013 24726877

[128] Lee SM Cheung CY Nicholls JM Hui KP Leung CY Uiprasertkul M. Hyperinduction of cyclooxygenase-2-mediated proinflammatory cascade: a mechanism for the pathogenesis of avian influenza H5N1 infection. J Infect Dis. (2008) 198:525–35. 10.1086/590499 18613795

[129] Carey MA Bradbury JA Rebolloso YD Graves JP Zeldin DC Germolec DR. Pharmacologic inhibition of COX-1 and COX-2 in influenza A viral infection in mice. PLoS ONE (2010) 5:e11610. 10.1371/journal.pone.0011610 20657653 PMC2904706

[130] Zheng BJ Chan KW Lin YP Zhao GY Chan C Zhang HJ. Delayed antiviral plus immunomodulator treatment still reduces mortality in mice infected by high inoculum of influenza A/H5N1 virus. Proc Natl Acad Sci USA. (2008) 105:8091–6. 10.1073/pnas.0711942105 18523003 PMC2430364

[131] Tacconelli S Capone ML Sciulli MG Ricciotti E Patrignani P. The biochemical selectivity of novel COX-2 inhibitors in whole blood assays of COX-isozyme activity. Curr Med Res Opin. (2002) 18:503–11. 10.1185/030079902125001335 12564662

[132] Starko KM. Salicylates and pandemic influenza mortality, 1918-1919 pharmacology, pathology, and historic evidence. Clin Infect Dis. (2009) 49:1405–10. 10.1086/606060 19788357

[133] Eyers S Weatherall M Shirtcliffe P Perrin K Beasley R. The effect on mortality of antipyretics in the treatment of influenza infection: systematic review and meta-analysis. J R Soc Med. (2010) 103:403–11. 10.1258/jrsm.2010.090441 20929891 PMC2951171

[134] Fedson DS. Pandemic influenza: a potential role for statins in treatment and prophylaxis. Clin Infect Dis. (2006) 43:199–205. 10.1086/505116 16779747 PMC7107836

[135] Mohammad S Nguyen H Nguyen M Abdel-Rasoul M Nguyen V Nguyen CD. Pleotropic Effects of Statins: untapped potential for statin pharmacotherapy. Curr Vasc Pharmacol. (2018). [Epub ahead of print]. 10.2174/1570161116666180723120608 30033872

[136] Haidari M Ali M Casscells SW Madjid M Statins block influenza infection by down-regulating rho/rho kinase pathway. Circulation (2007) 116:116–7. 10.1161/circ.116.suppl_16.II_7

[137] Mehrbod P Omar AR Hair-Bejo M Haghani A Ideris A. Mechanisms of action and efficacy of statins against influenza. Biomed Res Int. (2014) 2014:872370. 10.1155/2014/872370 25478576 PMC4244940

[138] Mehrbod P El Zowalaty M Omar AR Hair-Bejo M Ideris A. Statins reduce the expression of proinflammatory cytokines in influenza A virus infected CrFK cells. Acta Virol. (2012) 56:353–5. 10.4149/av_2012_04_353 23237094

[139] Lee CS Yi EH Lee JK Won C Lee YJ Shin MK. Simvastatin suppresses RANTES-mediated neutrophilia in polyinosinic-polycytidylic acid-induced pneumonia. Eur Respir J. (2013) 41:1147–56. 10.1183/09031936.00050612 22835616

[140] An SC Xu LL Li FD Bao LL Qin C Gao ZC. Triple combinations of neuraminidase inhibitors, statins and fibrates benefit the survival of patients with lethal avian influenza pandemic. Med Hypotheses (2011) 77:1054–7. 10.1016/j.mehy.2011.09.001 21944947

[141] Liu Z Guo Z Wang G Zhang D He H Li G. Evaluation of the efficacy and safety of a statin/caffeine combination against H5N1, H3N2 and H1N1 virus infection in BALB/c mice. Eur J Pharm Sci. (2009) 38:215–23. 10.1016/j.ejps.2009.07.004 19616097

[142] Kumaki Y Morrey JD Barnard DL. Effect of statin treatments on highly pathogenic avian influenza H5N1, seasonal and H1N1pdm09 virus infections in BALB/c mice. Future Virol. (2012) 7:801–18. 10.2217/fvl.12.71 23420457 PMC3571729

[143] Radigan KA Urich D Misharin AV Chiarella SE Soberanes S Gonzalez A. The effect of rosuvastatin in a murine model of influenza A infection. PLoS ONE (2012) 7:e35788. 10.1371/journal.pone.0035788 22536437 PMC3335012

[144] Enserink M. Infectious disease. Old drugs losing effectiveness against flu; could statins fill gap? Science (2005) 309:1976–7. 10.1126/science.309.5743.1976a 16179440

[145] Frost FJ Petersen H Tollestrup K Skipper B. Influenza and COPD mortality protection as pleiotropic, dose-dependent effects of statins. Chest (2007) 131:1006–12. 10.1378/chest.06-1997 17426203

[146] Carrillo-Esper R Sosa-Garcia JO Arch-Tirado E. Experience in the management of the severe form of human influenza A H1N1 pneumonia in an intensive care unit. Cir Cir. (2011) 79:409–16. 22385760

[147] Vandermeer ML Thomas AR Kamimoto L Reingold A Gershman K Meek J. Association between use of statins and mortality among patients hospitalized with laboratory-confirmed influenza virus infections: a multistate study. J Infect Dis. (2012) 205:13–9. 10.1093/infdis/jir695 22170954

[148] Kwong JC Li P Redelmeier DA. Influenza morbidity and mortality in elderly patients receiving statins: a cohort study. PLoS ONE (2009) 4:e8087. 10.1371/journal.pone.0008087 19956645 PMC2778952

[149] Fleming DM Verlander NQ Elliot AJ Zhao H Gelb D Jehring D. An assessment of the effect of statin use on the incidence of acute respiratory infections in England during winters 1998-1999 to 2005-2006. Epidemiol Infect. (2010) 138:1281–8. 10.1017/S0950268810000105 20109259

[150] Grygiel-Gorniak B. Peroxisome proliferator-activated receptors and their ligands: nutritional and clinical implications–a review. Nutr J. (2014) 13:17. 10.1186/1475-2891-13-17 24524207 PMC3943808

[151] Herold S Steinmueller M Von Wulffen W Cakarova L Pinto R Pleschka S. Lung epithelial apoptosis in influenza virus pneumonia: the role of macrophage-expressed TNF-related apoptosis-inducing ligand. J Exp Med. (2008) 205:3065–77. 10.1084/jem.20080201 19064696 PMC2605231

[152] Aldridge JRJr Moseley CE Boltz DA Negovetich NJ Reynolds C Franks J. TNF/iNOS-producing dendritic cells are the necessary evil of lethal influenza virus infection. Proc Natl Acad Sci USA. (2009) 106:5306–11. 10.1073/pnas.0900655106 19279209 PMC2664048

[153] Moseley CE Webster RG Aldridge JR. Peroxisome proliferator-activated receptor and AMP-activated protein kinase agonists protect against lethal influenza virus challenge in mice. Influenza Other Respir Viruses (2010) 4:307–11. 10.1111/j.1750-2659.2010.00155.x 20716159 PMC3584640

[154] Cloutier A Marois I Cloutier D Verreault C Cantin AM Richter MV. The prostanoid 15-deoxy-Delta12,14-prostaglandin-j2 reduces lung inflammation and protects mice against lethal influenza infection. J Infect Dis. (2012) 205:621–30. 10.1093/infdis/jir804 22219346

[155] Bottcher-Friebertshauser E Freuer C Sielaff F Schmidt S Eickmann M Uhlendorff J. Cleavage of influenza virus hemagglutinin by airway proteases TMPRSS2 and HAT differs in subcellular localization and susceptibility to protease inhibitors. J Virol. (2010) 84:5605–14. 10.1128/JVI.00140-10 20237084 PMC2876594

[156] Malakhov MP Aschenbrenner LM Smee DF Wandersee MK Sidwell RW Gubareva LV. Sialidase fusion protein as a novel broad-spectrum inhibitor of influenza virus infection. Antimicrob Agents Chemother. (2006) 50:1470–9. 10.1128/AAC.50.4.1470-1479.2006 16569867 PMC1426979

[157] Triana-Baltzer GB Babizki M Chan MC Wong AC Aschenbrenner LM Campbell ER. DAS181, a sialidase fusion protein, protects human airway epithelium against influenza virus infection: an *in vitro* pharmacodynamic analysis. J Antimicrob Chemother. (2010) 65:275–84. 10.1093/jac/dkp421 19942616 PMC2809246

[158] Triana-Baltzer GB Sanders RL Hedlund M Jensen KA Aschenbrenner LM Larson JL. Phenotypic and genotypic characterization of influenza virus mutants selected with the sialidase fusion protein DAS181. J Antimicrob Chemother. (2011) 66:15–28. 10.1093/jac/dkq387 21097900 PMC3001847

[159] Chan RW Chan MC Wong AC Karamanska R Dell A Haslam SM. DAS181 inhibits H5N1 influenza virus infection of human lung tissues. Antimicrob Agents Chemother (2009) 53:3935–41. 10.1128/AAC.00389-09 19596886 PMC2737896

[160] Triana-Baltzer GB Gubareva LV Klimov AI Wurtman DF Moss RB Hedlund M. Inhibition of neuraminidase inhibitor-resistant influenza virus by DAS181, a novel sialidase fusion protein. PLoS ONE (2009a) 4:e7838. 10.1371/journal.pone.0007838 19893749 PMC2770896

[161] Triana-Baltzer GB Gubareva LV Nicholls JM Pearce MB Mishin VP Belser JA. Novel pandemic influenza A(H1N1) viruses are potently inhibited by DAS181, a sialidase fusion protein. PLoS ONE (2009b) 4:e7788. 10.1371/journal.pone.0007788 19893747 PMC2770640

[162] Belser JA Lu X Szretter KJ Jin X Aschenbrenner LM Lee A. DAS181, a novel sialidase fusion protein, protects mice from lethal avian influenza H5N1 virus infection. J Infect Dis. (2007) 196:1493–9. 10.1086/522609 18008229

[163] Marjuki H Mishin VP Chesnokov AP De La Cruz JA Fry AM Villanueva J. An investigational antiviral drug, DAS181, effectively inhibits replication of zoonotic influenza A virus subtype H7N9 and protects mice from lethality. J Infect Dis. (2014) 210:435–40. 10.1093/infdis/jiu105 24569063 PMC4091581

[164] Zenilman JM Fuchs EJ Hendrix CW Radebaugh C Jurao R Nayak SU. Phase 1 clinical trials of DAS181, an inhaled sialidase, in healthy adults. Antiviral Res. (2015) 123:114–9. 10.1016/j.antiviral.2015.09.008 26391974 PMC4639451

[165] Moss RB Hansen C Sanders RL Hawley S Li T Steigbigel RT. A phase II study of DAS181, a novel host directed antiviral for the treatment of influenza infection. J Infect Dis. (2012) 206:1844–51. 10.1093/infdis/jis622 23045618 PMC3570175

[166] Siegel SJ Roche AM Weiser JN. Influenza promotes pneumococcal growth during coinfection by providing host sialylated substrates as a nutrient source. Cell Host Microbe (2014) 16:55–67. 10.1016/j.chom.2014.06.005 25011108 PMC4096718

[167] Hedlund M Aschenbrenner LM Jensen K Larson JL Fang F. Sialidase-based anti-influenza virus therapy protects against secondary pneumococcal infection. J Infect Dis. (2010) 201:1007–15. 10.1086/651170 20170378 PMC2874251

[168] Kotenko SV Gallagher G Baurin VV Lewis-Antes A Shen M Shah NK. IFN-lambdas mediate antiviral protection through a distinct class II cytokine receptor complex. Nat Immunol. (2003) 4:69–77. 10.1038/ni875 12483210

[169] Sheppard P Kindsvogel W Xu W Henderson K Schlutsmeyer S Whitmore TE. IL-28, IL-29 and their class II cytokine receptor IL-28R. Nat Immunol. (2003) 4:63–8. 10.1038/ni873 12469119

[170] Crotta S Davidson S Mahlakoiv T Desmet CJ Buckwalter MR Albert ML. Type I and type III interferons drive redundant amplification loops to induce a transcriptional signature in influenza-infected airway epithelia. PLoS Pathog. (2013) 9:e1003773. 10.1371/journal.ppat.1003773 24278020 PMC3836735

[171] Sommereyns C Paul S Staeheli P Michiels T. IFN-lambda (IFN-lambda) is expressed in a tissue-dependent fashion and primarily acts on epithelial cells *in vivo*. PLoS Pathog. (2008) 4:e1000017. 10.1371/journal.ppat.1000017 18369468 PMC2265414

[172] Galani IE Triantafyllia V Eleminiadou EE Koltsida O Stavropoulos A Manioudaki M. Interferon-lambda mediates non-redundant front-line antiviral protection against influenza virus infection without compromising host fitness. Immunity (2017). 46:875–90.e6. 10.1016/j.immuni.2017.04.025 28514692

[173] Klinkhammer J Schnepf D Ye L Schwaderlapp M Gad HH Hartmann R. IFN-lambda prevents influenza virus spread from the upper airways to the lungs and limits virus transmission. Elife (2018) 7:e33354. 10.7554/eLife.33354 29651984 PMC5953542

[174] Kim S Kim MJ Kim CH Kang JW Shin HK Kim DY. The superiority of IFN-lambda as a therapeutic candidate to control acute influenza viral lung infection. Am J Respir Cell Mol Biol. (2017) 56:202–12. 10.1165/rcmb.2016-0174OC 27632156

[175] Muir AJ Arora S Everson G Flisiak R George J Ghalib R. A randomized phase 2b study of peginterferon lambda-1a for the treatment of chronic HCV infection. J Hepatol. (2014) 61:1238–46. 10.1016/j.jhep.2014.07.022 25064437

[176] Topham DJ Tripp RA Doherty PC. CD8+ T cells clear influenza virus by perforin or Fas-dependent processes. J Immunol. (1997) 159:5197–200. 9548456

[177] Fujimoto I Takizawa T Ohba Y Nakanishi Y. Co-expression of Fas and Fas-ligand on the surface of influenza virus-infected cells. Cell Death Differ. (1998) 5:426–31. 10.1038/sj.cdd.4400362 10200492

[178] Ishikawa E Nakazawa M Yoshinari M Minami M. Role of tumor necrosis factor-related apoptosis-inducing ligand in immune response to influenza virus infection in mice. J Virol. (2005) 79:7658–63. 10.1128/JVI.79.12.7658-7663.2005 15919918 PMC1143624

[179] Brincks EL Katewa A Kucaba TA Griffith TS Legge KL. CD8 T cells utilize TRAIL to control influenza virus infection. J Immunol. (2008a) 181:4918–25. 10.4049/jimmunol.181.7.4918 18802095 PMC2610351

[180] Brincks EL Gurung P Langlois RA Hemann EA Legge KL Griffith TS. The magnitude of the T cell response to a clinically significant dose of influenza virus is regulated by TRAIL. J Immunol. (2011) 187:4581–8. 10.4049/jimmunol.1002241 21940678 PMC3197947

[181] Kash JC Tumpey TM Proll SC Carter V Perwitasari O Thomas MJ. Genomic analysis of increased host immune and cell death responses induced by 1918 influenza virus. Nature (2006) 443:578–81. 10.1038/nature05181 17006449 PMC2615558

[182] Brincks EL Kucaba TA Legge KL Griffith TS. Influenza-induced expression of functional tumor necrosis factor-related apoptosis-inducing ligand on human peripheral blood mononuclear cells. Hum Immunol. (2008b) 69:634–46. 10.1016/j.humimm.2008.07.012 18723061 PMC2597454

[183] Hogner K Wolff T Pleschka S Plog S Gruber AD Kalinke U. Macrophage-expressed IFN-beta contributes to apoptotic alveolar epithelial cell injury in severe influenza virus pneumonia. PLoS Pathog. (2013) 9:e1003188. 10.1371/journal.ppat.1003188 23468627 PMC3585175

[184] Fujikura D Chiba S Muramatsu D Kazumata M Nakayama Y Kawai T. Type-I interferon is critical for FasL expression on lung cells to determine the severity of influenza. PLoS ONE (2013) 8:e55321. 10.1371/journal.pone.0055321 23408968 PMC3568138

[185] Shim JM Kim J Tenson T Min JY Kainov DE. Influenza virus infection, interferon response, viral counter-response, and apoptosis. Viruses (2017) 9:223. 10.3390/v9080223 28805681 PMC5580480

[186] Bulanova D Ianevski A Bugai A Akimov Y Kuivanen S Paavilainen H. Antiviral Properties of chemical inhibitors of cellular anti-apoptotic Bcl-2 proteins. Viruses (2017) 9:271. 10.3390/v9100271 28946654 PMC5691623

[187] Kakkola L Denisova OV Tynell J Viiliainen J Ysenbaert T Matos RC. Anticancer compound ABT-263 accelerates apoptosis in virus-infected cells and imbalances cytokine production and lowers survival rates of infected mice. Cell Death Dis. (2013) 4:e742. 10.1038/cddis.2013.267 23887633 PMC3730437

